# Drug Discovery Study Aimed at a Functional Cure for HBV

**DOI:** 10.3390/v14071393

**Published:** 2022-06-26

**Authors:** Takehisa Watanabe, Sanae Hayashi, Yasuhito Tanaka

**Affiliations:** Department of Gastroenterology and Hepatology, Faculty of Life Sciences, Kumamoto University, Kumamoto 860-8556, Japan; twatanabe@kumadai.jp (T.W.); sanaehayashi66@gmail.com (S.H.)

**Keywords:** HBV, functional cure, HBV-RNA, HBcrAg, iTACT-HBcrAg

## Abstract

Hepatitis B virus (HBV) causes acute and, most importantly, chronic hepatitis B worldwide. Antiviral treatments have been developed to reduce viral loads but few patients with chronic hepatitis B (CHB) achieve a functional cure. The development of new therapeutic agents is desirable. Recently, many novel agents have been developed, including drugs targeting HBV-DNA and HBV-RNA. This review provides an overview of the developmental status of these drugs, especially direct acting antiviral agents (DAAs). Serological biomarkers of HBV infection are essential for predicting the clinical course of CHB. It is also important to determine the amount and activity of covalently closed circular DNA (cccDNA) in the nuclei of infected hepatocytes. Hepatitis B core-associated antigen (HBcrAg) is a new HBV marker that has an important role in reflecting cccDNA in CHB, because it is associated with hepatic cccDNA, as well as serum HBV DNA. The highly sensitive HBcrAg (iTACT-HBcrAg) assay could be a very sensitive HBV activation marker and an alternative to HBV DNA testing for monitoring reactivation. Many of the drugs currently in clinical trials have shown efficacy in reducing hepatitis B surface antigen (HBsAg) levels. Combination therapies with DAAs and boost immune response are also under development; finding the best combinations will be important for therapeutic development.

## 1. Introduction

Hepatitis B is an significant infectious disease, with a similar infection rate to human immunodeficiency virus (HIV) infection, tuberculosis, and malaria. It is estimated that there are currently about 300 million hepatitis B virus (HBV) carriers worldwide, with an annual death toll of about 1 million (World Health Organization, https://www.who.int/news-room/fact-sheets/detail/hepatitis-b (accessed on 20 May 2022)).

To eliminate hepatitis B, a vaccine is needed first to prevent vertical and horizontal transmission. In addition, treatment with interferon (IFN)/Peg-IFN, lamivudine (LAM), adefovir (ADF), entecavir (ETV), and tenofovir (TDF)/tenofovir alafenamide (TAF) has been used in developed countries to suppress HBV replication and cure chronic hepatitis B (CHB). Such approaches have made it possible to control the disease of hepatitis B. However, few patients are able to achieve a functional cure (hepatitis B surface antigen (HBsAg) negative), which is the current goal of treatment for CHB. Furthermore, therapeutic agents that do not belong to the categories of nucleos(t)ide reverse transcriptase inhibitors (nucleoside analogues; NAs) and IFNs have not been marketed for a long time, and the development of new therapeutic methods is desired.

Serological biomarkers of HBV are very important for predicting the course of CHB and are clinically useful as an alternative to invasive liver biopsy. On the other hand, it is important to determine the amount and activity of covalently closed circular DNA (cccDNA) present in the nuclei of infected hepatocytes, because this is difficult to eliminate. However, direct testing for cccDNA requires invasive liver biopsies. Hepatitis B core-associated antigen (HBcrAg) is a new HBV marker that has a pivotal role in assessing cccDNA in CHB because it is associated with hepatic cccDNA as well as serum HBV DNA. In addition, the highly sensitive HBcrAg (iTACT-HBcrAg) assay may be a very sensitive HBV activation marker and an alternative to HBV DNA testing for monitoring reactivation. Because many of the anti-HBV drugs currently in development are based on the concomitant use of NAs, HBV DNA assays are not suitable for monitoring and determining therapeutic efficacy during development, but HBcrAg will surely be useful.

This review focuses on and outlines new therapies that target intrahepatic HBV replication. In particular, the latest findings on drugs targeting HBV nucleic acids are described. The role of serum HBcrAg testing in the treatment of CHB also is discussed.

## 2. Drug Discovery Study Aimed at HBV

### 2.1. Current HBV Treatment and Problems

NAs and Peg-IFN are approved antiviral therapies against HBV, aimed at lowering or eliminating HBsAg and cccDNA.

NAs such as LAM, ADF, ETV, and TDF/TAF inhibit reverse transcription (inhibit HBV DNA replication) and can reduce HBV DNA and ALT levels at a high rate but are rarely expected to reduce HBsAg or cccDNA. Another problem with NAs is the possibility that resistance may emerge with long-term use.

On the other hand, IFN/Peg-IFN therapy has a variety of action points, mainly immunostimulatory effects, and can induce HBsAg negativity, but the success rate of treatment is very low and adverse events are common. It has also been pointed out that treatment may induce few HBV-specific immune responses [1].

Recently, it was reported that when TDF, an NA, was combined with Peg-IFN and administered to untreated patients, a higher rate of negative serum HBsAg was observed than with Peg-IFN alone (IFN alone: 2.8%, TDF + IFN combination: 9.1% at 72 weeks after completion of treatment) [2]. However, add-on therapy with Peg-IFN in long-term NA patients was subsequently investigated but did not lower HBsAg [3]. Therefore, it appears that these current therapies alone are not sufficient to eliminate HBV.

### 2.2. Development of Drugs Aimed at a Functional Cure

Since it is unlikely that HBV can be completely eliminated with current therapies, the development of novel agents with mechanisms that differ from those current therapies is underway worldwide [4].

Specifically, there are two main approaches aiming to eliminate HBV: (1) direct-acting antiviral agents (DAAs), which target the replication cycle and directly inhibit HBV and (2) drugs that act on host factors, mainly inducing HBV-specific immune responses. This review focuses on DAAs under clinical trials, especially those targeting HBV nucleic acids.

**Figure 1:**
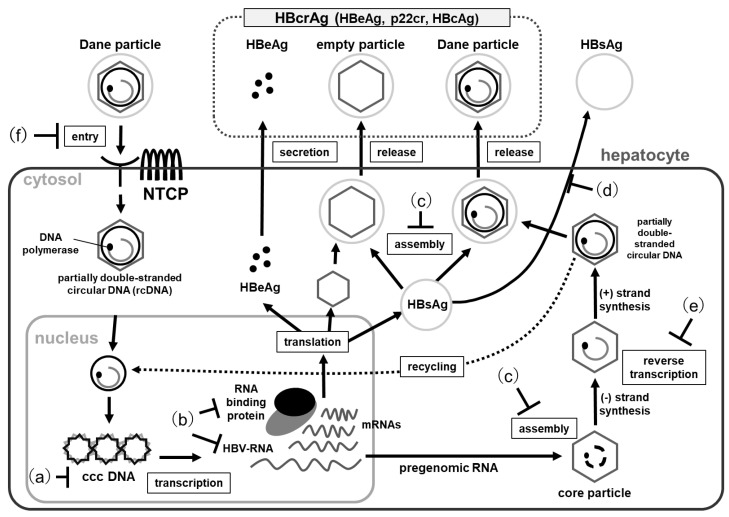
Replication cycle of HBV and steps targeted by therapeutic agents (DAAs). (**a**) Targeting cccDNA, (**b**) Targeting viral RNA or HBV RNA binding protein, (**c**) Inhibition of capsid formation or nucleocapsid assembly, (**d**) HBsAg Inhibitors, (**e**) Inhibition of reverse transcription. (**f**) Inhibition of HBV entry into hepatocytes.

Therapeutic direct-acting antiviral agents for HBV in the liver are roughly as shown in Figure 1: (a) Targeting cccDNA, converted from genomic DNA by covalent binding in the cell nucleus. This cccDNA contributes to persistent viral infection and is the most important factor preventing viral elimination by therapy. (b) Targeting HBV RNA, transcribed using cccDNA as a template. HBV RNA behaves similar to mRNA, which is translated into viral proteins, and pre-genomic RNA (pgRNA), which is reverse transcribed into HBV genomic DNA in the nucleocapsid. (c) Targeting viral nucleocapsid assembly, formed from core protein and encasing the pgRNA and polymerase. (d) Targeting HBsAg and (e) HBV polymerase inhibitors. (f) Entry inhibitors, targeting the process by which HBV adsorbs to and invades hepatocytes via receptors. In this section, we will focus on current treatments for CHB and a few new potential therapeutic agents, particularly as they relate to their effects on the intrahepatic replication cycle of HBV (Table 1).

### 2.3. HBV Therapeutic Drug Development Targeting HBV DNA and HBV RNA

The HBV genome exists as cccDNA in the hepatocyte nucleus, from which are transcribed a 3.5 kb mRNA that functions as a pregenomic RNA, 2.4 kb and 2.1 kb mRNAs that encode HBsAg and a 0.7 kb mRNA that encodes the HBx protein (Figure 1). The RNA inhibitors (A) small interfering RNAs (siRNA) and (B) anti-sense oligonucleotides (ASO) are drugs that inhibit these viral RNAs and are used in clinical trials. In addition, a new class of drugs, (C) RNA-binding protein inhibitors, is under development. These drugs aim to suppress the production of HBsAg and achieve a functional cure.

### 2.4. (A) siRNA Targeting HBV RNA

siRNA is a small double-stranded RNA consisting of 21–23 bases. siRNA directly acts on the target RNA by RNA interference (RNAi) to induce its degradation and inhibit the expression of the encoded protein. The siRNA, together with the RNA-induced silencing complex (RISC), binds to and induces cleavage of the HBV RNA, thereby exerting a potent HBsAg-lowering effect. In experiments using chimeric mice with human livers, the siRNA reduced the concentration of HBV DNA and HBsAg in serum to 1/10 or less [5]. Currently, several siRNA drugs targeting HBV RNA are under development (Table 1). ARC-520 is the first siRNA to show positive results in a phase I study in CHB patients, including the disappearance of HBsAg from some patients [6]. However, the development of ARC-520 was discontinued, as additional animal studies showed toxicity at high doses. Subsequently, ARO-HBV (JNJ-3989), a drug-treated with N-acetylgalactosamine (N-acetylgalactosamine; GalNAc), a liver-directed drug delivery system (DDS), was developed.

GalNAc binds to asialoglycoproteins expressed on the surfaces of hepatocytes, allowing efficient liver uptake of target molecules. While ARC-520 was administered intravenously, JNJ-3989 can be administered subcutaneously. In a phase I/II clinical trial in combination with NA, monthly administration of JNJ-3989 resulted in a decrease in serum HBsAg of at least 1 log IU/mL in all patients, with 88% of patients having HBsAg levels below 100 IU/mL. Furthermore, an average HBsAg reduction of more than 1 log IU/mL was maintained for six months [7]. It is of interest to see whether this sustained HBsAg reduction is accompanied by an immune-inducing effect, as well as a direct anti-HBV effect by siRNA. JNJ-3989 and most of the siRNA formulations currently in phase II trials (VIR-2218, RG-6346, AB-729, etc.) have shown potent HBsAg-lowering effects, reducing HBsAg by more than 1 log IU/mL in 85–92% of subjects and more than 50% of subjects achieved an HBsAg level below 100 IU/mL. In addition, a Phase IIb study of a three-drug combination therapy consisting of JNJ-3989, NA, and a capsid assembly inhibitor (JNJ-6379) is currently underway with the participation of more than 15 countries, including Japan (Reef-1 Study).

### 2.5. (B) Antisense Oligonucleotides (ASO)

Antisense oligonucleotides (ASOs) are short single-stranded DNA or RNA molecules, less than 20 bases long, that are complementary to the target sequence. They regulate RNA function by binding to the RNA. Antisense DNA binds complementary RNA, and the complex of the DNA/RNA pair that is formed is rapidly degraded by ribonuclease H (RNase H1).

ASOs use different combinations of DNA and modified nucleic acids and act in a variety of ways, including targeting all types of RNA and limiting their action to mRNA precursors and miRNAs [8,9]. In addition, by designating the target of the modified nucleic acid as a regulatory sequence of RNA, they can exert various effects, such as suppressing or enhancing the function of RNA and making it possible to develop drugs for various pathological conditions.

Liver-directed ASOs designed for anti-HBV therapy aim to degrade viral RNA to block the expression of viral proteins. Using 8- to 10-base DNA strands modified to resist nucleases, ASO binds to and cleaves through RNase H1, the nuclear and cytoplasmic HBV RNA transcribed from HBV cccDNA, thereby inhibiting translation of the viral protein and demonstrating antiviral activity. In particular, it inhibits the synthesis of not only pgRNA but also HBsAg, which cannot be targeted by NAs, and is thus expected to reduce HBsAg and induce a functional cure.

GSK3389404, an ASO binding to GalNAc, is taken up by the liver via asialoglycoprotein, acting in a liver-specific manner. Initially, a trial was conducted with GSK3389404, but due to insufficient anti-HBV efficacy, a Phase II trial of GSK3228836 (beprovirsen) without GalNAc is currently underway. GSK3228836, administered subcutaneously twice a week at the beginning and once a week from the third week, significantly reduced HBsAg [10]. Especially in some cases of the 300 mg group, HBsAg was below the limit of quantification, and two of the patients experienced long-term HBsAg loss. Most adverse events with this drug, such as redness at the injection site during administration, are mild/moderate in severity and the drug is considered to be safe [11]. Although this drug is a potent agent that can induce HBsAg loss, it is necessary to examine how much sustained HBsAg loss can be achieved after completion of dosing. Sequential therapy from GSK3228836 to PEG-IFN is also in phase II trials.

### 2.6. (C) Problems with Nucleic Acid Drugs

Nucleic acid drugs such as siRNA and ASOs are attractive modalities because of their different approaches compared to conventional small molecule drugs. For example, beprovirsen is assumed to reduce all transcripts from cccDNA by targeting common sequences in all HBV mRNAs and pgRNA [10]. On the other hand, they have diverse mechanisms of toxicity because of the difference. In particular, sequence-dependent off-target effects should not occur. In common with nucleic acid drugs, thrombocytopenia, hepatorenal toxicity, and toxicity due to immune response appear relatively frequently, and these class effects need to be improved. The possibility of the emergence of anti-nucleic acid drug antibodies has also been suggested, and the effects of the antibodies that may arise need to be considered.

### 2.7. (D) Drugs Targeting RNA Binding Protein

RG-7834 is a novel oral HBV antiviral drug belonging to the dihydroquinolizinones (DHQ) class, which acts as an RNA destabilizer by inhibiting the RNA-binding protein PAPD5/7, destabilizing HBV RNA, and promoting its degradation [12]. RG-7834 has been shown to selectively inhibit HBV transcription in HBV-infected human liver chimeric uPA/SCID (PXB) mice. Although the development of RG-7834 has been suspended due to undisclosed adverse events, studies are underway to combine it with a liver-specific DDS to circumvent its effects on other organs [13].

The new compound we are developing belongs to the RNA destabilizer class and is a promising drug with potent HBsAg-lowering activity and no apparent adverse events [14].

### 2.8. Targeting HBV cccDNA for Cure of Chronic Hepatitis B

Following HBV infection of hepatocytes, the virus forms cccDNA in the nucleus, which remains stable as a persistent reservoir of HBV replication [15,16,17]. It remains unclear how the formation and maintenance of intrahepatic cccDNA is regulated in the persistence of HBV infection, but cccDNA regulation is believed to be a complex process involving host and/or viral factors [18,19,20,21,22,23]. Intrahepatic cccDNA levels in patients with CHB range from 0.035 to 195 copies/cell, and these levels are lower in HBeAg-negative patients and inactive carriers than HBeAg-positive patients [24,25]. On the other hand, cccDNA remains in the hepatocytes of patients after HBV treatment, potentially causing HBV reactivation.

Currently used therapeutic agents for CHB, Peg-IFN and NAs do not directly target cccDNA, making it difficult to achieve a “functional cure” [25,26]. Therefore, the complete eradication of HBV infection requires the removal of cccDNA by new methods that act directly or indirectly on HBV [27]. Methods for cleavage and removal of HBV cccDNA by gene-editing nucleases are under development, including zinc finger nuclease (ZFN) [28,29], transcription activator-like effector nuclease (TALEN) [30,31], and cluster-regular interspace short palindromic repeat-CAS9(CRISPR/CAS9) [32,33,34,35,36,37].

The CRISPR/Cas9 system is a programmed genome that can specifically edit DNA sequences by manipulating guide RNAs specific to target sequences and has attracted attention due to its simpler design and lower costs than ZFN and TALEN [37,38]. It has been reported that CRISPR/Cas9 cccDNA cleavage inhibits HBV replication and the synthesis of HBcrAg and HBsAg in vitro and in vivo [32,37,39].

Interestingly, the CRISPR/Cas9 system has been shown to reduce cccDNA by up to 92% in vitro [35]. In particular, Karimova et al. found a specific site in the S and X regions of the HBV genome, the targeting of which by the Cas9 system disrupted not only the integration of HBV sequences and episomal cccDNA in the reporter HEK293 stable cell line, but also HBV replication in chronically and de novo infected cell lines [40]. More recently, Stones et al. showed that cccDNA in chronic infection with HBV in PXB mice was reduced by adeno-associated virus (AAV) vectors and CRISPR/Cas9 [41]. More recently, Yang et al. demonstrated that CRISPR/Cas9-mediated “base editors” (BEs) significantly reduce the expression of HBsAg by generating nonsense mutations in the HBV surface open reading frame [42]. The CRISPR/Cas9-mediated non-cleavage BEs are expected to generate more permanent inactivation of the integrated HBV genome and cccDNA than siRNA-based strategies. These findings suggest that CRISPR/Cas9 may be a novel therapeutic approach for the treatment of both chronic HBV infection and de novo HBV infection. The greatest advantage of gene editing is the ability to completely eliminate HBV from hepatocytes by disrupting cccDNA. On the other hand, the disadvantage is that off-targets may occur. Genome-editing therapies are still in a proof-of-concept stage and challenging before their clinical application. The key challenges are (1) to improve upon the specificity [43], (2) to target HBV mutations and various HBV genotypes [44,45], (3) avoid undesirable effects [46,47], and (4) efficiently deliver CRISPR/Cas9 to HBV-infected cells [48]. Notably, the HBx/DNA damage-binding protein 1 (DDB1) complex promotes transcription of pgRNA from cccDNA [49]. Nitazoxanide (NTZ), a thiazolidine for protozoan enteritis, was identified as an inhibitor of the HBx-DDB1 interaction, resulting in efficient inhibition of the transcription of cccDNA and reduction in cccDNA in vitro with a promising new therapeutic option [50].

Recent studies have estimated the half-life of cccDNA to be several months in NAs-suppressed CHB patients [51,52], suggesting that potent NAs inhibit new rcDNA production, completely block cccDNA replenishment and may promote cccDNA reduction [26,52,53,54,55,56,57,58,59]. Currently, ETV, TDF, and TAF are recommended for the treatment of CHB patients due to their high efficacy at controlling HBV infection, such as normalization of ALT, HBV DNA suppression and prevention of liver progressions. However, the emergence of antiviral-resistant mutations in the RT region is a disadvantage of lifelong treatment for CHB patients. Recently, Kuwata et al. identified a novel NA, (1S,3S,5S,E)-3-(2-Amino-6-oxo-1,6-dihydro-9H-purin-9-yl)-2-(fluoromethylene)-5-hydroxy-1-(hydroxy-methyl)cyclopentane-1-carbonitrile(*E*-CFCP) [60]. *E*-CFCP had potent activity against wild-type and drug-resistant mutants, inhibited HBV DNA synthesis more completely with longer anti-HBV activity after administration and showed less toxicity than ETV and TAF [60]. These data suggest that *E*-CFCP promises to reduce the emergence of drug resistance and improve treatment compliance and quality of life. If further studies reveal that long-acting *E*-CFCP with potent activity against HBV accelerates the reduction in intrahepatic cccDNA, it would be expected to improve preparedness for the functional cures in CHB patients with current treatment with NAs. The steady-state levels of cccDNA are determined by the rate of its generation and loss, but how the cccDNA molecules survive mitosis (i.e., whether cccDNA is randomly or evenly distributed between daughter cells) remains unclear [61,62]. Therefore, elucidating the molecular mechanism of cccDNA formation will enable the development of therapeutic agents that can achieve a functional cure and of new therapeutic applications in the future [63].

### 2.9. Entry Inhibitors

Myrcludex B, an entry inhibitor, has been reported to be a strong competitive inhibitor of the binding of HBV to NTCP, the receptor for HBV on hepatocytes, and clinical trials are underway. At the end of 24 weeks of treatment, Myrcludex B alone did not reduce HBV DNA in serum, but the combination group of Myrcludex B and Peg-IFN showed stronger HBV DNA reduction than Peg-IFN alone [64]. However, since there was no significant difference in the serum HBsAg levels between the two groups in this study, the effect of long-term (48 weeks) combination treatment with Myrcludex B and Peg-IFN on HBsAg is currently being investigated. On the other hand, Myrcludex B administration does not cause serious adverse events but may cause inhibition of bile acid uptake due to the competitive inhibition of NTCP. Therefore, basic research on agents that selectively inhibit HBV adsorption without inhibiting NTCP transporter activity, and that inhibit intracellular entry of HBV, has recently been conducted [65,66].

### 2.10. Capsid Assembly Inhibitors (CAMs)

The core (HBc) protein quickly dimerizes after translation from mRNA, and then the dimers assemble to form a capsid. Inside the capsid, the pgRNA interacts with reverse transcriptase and reverse transcription occurs. When nucleocapsid formation is inhibited, reverse transcription from pgRNA to rcDNA does not occur and HBV replication is suppressed. Capsid assembly inhibitors (CAMs) inhibit the formation of the nucleocapsid and reverse transcription of pgRNA to rcDNA, thereby suppressing HBV replication [67]. CAMs are promising drugs because they not only directly inhibit capsid formation in the liver but may also inhibit de novo synthesis of cccDNA. NAs inhibit the reverse transcription reaction that occurs after pgRNA is incorporated into the core particles. HBcrAg reflects the broad life cycle of HBV, including not only Dane particles, but also particles encapsulating HBV RNA, empty particles without HBV RNA, and HBe antigen. Thus, NAs reduce HBV DNA but do not affect HBV RNA or HBcrAg because immature HBV particles without DNA are released into the blood. On the other hand, CAMs have a broader effect on the HBV replication cycle because they inhibit capsid assembly, the site of the reverse transcription reaction, and reduce not only HBV DNA but also HBV RNA and HBcrAg. CAMs are broadly classified into two types: Class 1 inhibitors, which inhibit capsid formation and cause the formation of abnormal forms of the capsid, and Class 2 inhibitors, which inhibit pgRNA incorporation into the capsid. In the phase I study JNJ-6379, one of the drugs classified as class 2, produced a decrease in the serum HBV DNA level of −2.86 logIU/mL after 4 weeks of administration, but no change in HBsAg levels [68]. On the other hand, it has been reported that JNJ-6379 inhibits nuclear transfer of rcDNA and suppresses cccDNA formation and recycling in vitro, and further studies including long-term administration are expected [67].

### 2.11. Nucleic Acid Polymers (NAPs)

Nucleic acid polymers (NAPs) inhibit the secretion of HBsAg and protect against infection. A phase II study of two NAPs, REP2139 and REP2165 reported that 35% of patients with the three-drug combination of REP2139 or REP2165 plus TDF and Peg-IFN remained functionally cured and HBs antibodies appeared at 48 weeks after the end of treatment [69]. On the other hand, liver injury occurring during treatment is an issue that needs to be resolved. REP2139 has efficacy against not only HBV but also Hepatitis D virus and is currently in phase II clinical trials.

### 2.12. Drugs That Act on Host Immunity to Eliminate HBV

Experiments using chimpanzees have shown that immune responses, especially T cell responses, are important for HBV elimination [70]. While a large number of HBV-specific CD8+ T cells have been observed in patients who had acute hepatitis B and from whom HBV was eliminated, the number of such cells is markedly lower in patients with CHB who are persistently infected with HBV, and a functional decline in cytokine production capacity, including IFNγ, is observed. In this state, immune tolerance (immune exhaustion) PD-1 is highly expressed on T cells [71].

To boost the number of HBV-directed T cells, researchers are working on several immune-stimulating strategies. Agents that act on host factors to eliminate HBV include TLR agonists, therapeutic vaccines and checkpoint inhibitors. Therapeutic vaccines are the most popular agents among them.

TLRs are receptors expressed on the cell surface or within the endosomes of various cells. HBV is rarely recognized by innate immune sensors and has a low ability to induce innate immunity [72]. GS-9620, a TLR7 agonist, has been shown to reduce HBV DNA and HBsAg in serum in experiments using chimpanzees [73]. However, in clinical trials, this did not affect the amount of HBsAg in serum, although it was able to induce some functionality of CD8+ T cells and NK cells [74,75]. Currently, development and clinical trials of GS-9688, a TLR8 agonist, and AIC649, a TLR9 agonist, are underway.

Nasal vaccine candidate (NASVAC) is an intranasal therapeutic vaccine containing two antigens, HBsAg and HBcAg, and is intended to activate immune-depleted T cells or B cells.

A phase III trial was conducted in untreated patients in Bangladesh, comparing two courses of five doses of NASVAC with 48 weeks of Peg-IFN. The results showed the efficacy of NASVAC in lowering blood HBV DNA levels at 24 weeks after treatment [76].

Recently, nivolumab, an anti-PD-1 antibody, has been in the spotlight in the field of malignant tumors. In a phase Ib study of nivolumab in CHB patients, the serum HBsAg level decreased by an average of −0.48 log IU/mL at 24 weeks after a single dose of nivolumab, and one of 12 patients became HBsAg negative, but this negative case was accompanied by Grade 3 liver dysfunction [77].

These results suggest that further investigation of strategies to achieve more specific therapeutic effects is needed to reduce immune-related adverse effects (irAEs).

### 2.13. Clinical Utility of the Highly Sensitive HBcrAg Assay for Drug Development

HBcrAg consists of HBeAg, HBcAg in Dane particles, and a truncated 22 kDa precore protein (p22cr) in empty Dane-like particles [78]. All three proteins can be detected non-invasively as HBcrAg by serological examination [79,80]. The levels of HBcrAg reflect HBV DNA levels in serum and liver and cccDNA in the liver [81,82], suggesting the importance of HBcrAg measurement in CHB monitoring [79,82]. The current goal for patients with HBV infection is to achieve virological suppression and, ultimately, HBsAg serum clearance, resulting in biochemical remission, histological improvement, and a reduced risk of complications [81,83]. Since serum HBcrAg is correlated with intrahepatic cccDNA [82], HBcrAg has been considered as a serum marker for predicting viral recurrence (relapse) after stopping NAs therapy, as follows: NAs are potent viral replication inhibitors that block HBV reverse transcription, but HBcrAg remains in 78% of CHB patients who achieve undetectable serum HBV DNA, and HBcrAg levels gradually decrease during the treatment [82]. Serum HBcrAg levels can show a positive correlation with intrahepatic cccDNA levels, even in NAs-treated patients [51,84].

Furthermore, HBcrAg levels were reported to be a predictor of the risk of virological relapse that occurs when patients with undetectable serum HBV DNA discontinue NAs [85]. Furthermore, HBcrAg levels at NA discontinuation (<3.4 log U/mL) were the only independent predictor of no relapse. Among CHB patients with undetectable serum HBV DNA levels for the duration of NAs therapy, long-term monitoring of serum HBcrAg levels is suitable [82], and the decline pattern could reasonably provide predictive information about the incidence of HBV reactivation following the therapy [86].

The combination of HBcrAg and HBsAg levels at end-of-treatment (HBsAg <10 IU/mL and HBcrAg <2 log levels) is predictive of HBsAg loss [87] and the HBcrAg levels are useful in determining the efficacy of NA treatment for CHB patients [86], suggesting that the following iTACT-HBcrAg assay would be more useful. Serum HBcrAg may be predictive of HBeAg seroconversion and HBV DNA reduction for CHB patients during PEG-IFN treatment [88,89]. In HBeAg-positive patients, a high HBcrAg level (>8 logs U/mL) at baseline had a >94.4% negative predictive value (NPV) for achieving HBeAg seroconversion and HBV DNA suppression after 12 weeks of PEG-IFN therapy [88]. In 50 patients treated with PEG-IFN in combination with NAs for 1 month, followed by PEG-IFN for 5 months, baseline HBcrAg levels (>4.5 log U/mL) indicated no response and induced no HBeAg seroconversion at 2 years post-treatment [90]. Recently, Hayashi et al. demonstrated that the more profound reduction of cccDNA in the viral responder (VR) group than in the non-VR group was associated with higher peak ALT activities during PEG-IFN treatment, suggesting the role of cell death in the elimination of cccDNA [91]^.^ Additionally, the levels of serum HBcrAg correlate with the levels of cccDNA in the liver (r = 0.90849, *p* = 8.46 × 10^−10^). Therefore, monitoring the dynamic changes in HBcrAg during anti-viral therapy would be a predictor of clinical outcomes.

HBcrAg is also useful for evaluating the efficacy of new drugs under development. Several studies have reported that the kinetics of HBcrAg have been assessed in the investigation of several new anti-HBV compounds. Lin et al. reported that the CAM, BAY41–4109, reduced intrahepatic core proteins and de novo synthesis of cccDNA [92]. ARC-520, an siRNA, reduced serum HBcrAg by 1.4 logs kU/mL on day 85 by a single dose of intravenous for HBeAg-positive CHB [93]. Combination therapy of NAs and ARC-520 led to a reduction in HBV antigen production, including HBcrAg, in HBeAg-positive CHB patients [94]. The results of these studies suggest that HBcrAg could be a useful biomarker for monitoring the effectiveness of anti-HBV therapeutic targets and the development of new drugs brings new promises for curing HBV.

More recently, Inoue et al. have reported that a fully automated, high-sensitivity CLEIA for HBcrAg detection (iTACT-HBcrAg; Fujirebio Corporation, Tokyo, Japan) is useful for the management of HBV reactivation and of HBeAg-negative CHB patients [95,96]. The iTACT-HBcrAg assay is inexpensive and simpler to use than the HBV DNA assay, and provides results within 30 min. Furthermore, the iTACT-HBcrAg assay is approximately ten times more sensitive than conventional HBcrAg assays and may be useful in determining disease progression and prognosis, as described above for CHB patients [97]. In addition, because several new therapeutic agents under development may directly inhibit the expression of intracellular core proteins and eliminate cccDNA contents, monitoring by a more sensitive HBcrAg assay may be useful for determining the therapeutic effects [83]. Hence, this highly accurate assay could be more useful in clinical practice.

## 3. Discussion

### 3.1. Development of Useful Drugs for HBsAg Reduction

The goal of HBV treatment is to first achieve negative HBV DNA and then negative HBsAg to achieve a functional cure. NAs alone cannot achieve a functional cure, but the advantage of NAs is that they are well-tolerated with few adverse events. The treatment of chronic viral infections often involves multidrug combination therapy. The novel agents described in this review can lower HBsAg. We believe that combination therapy with NAs is the most realistic way to achieve a functional cure. A possible combination to completely inhibit hepatic HBV replication would consist of an NA and one or two other direct-acting antiviral agents with different mechanisms, such as CAM, siRNA, cccDNA inhibitors, and entry inhibitors. Currently, multidrug combination therapy, combining two or more agents, is under development. The three-drug combination of RNAi (JNJ-3989) + CAM (JNJ-6379) + NA has shown significant inhibition of production of HBV DNA, HBV RNA, HBsAg and HBcrAg, and is well tolerated with no serious adverse events reported [98].

Prolonged exposure to high viral antigen levels can exhaust the host immune response and lead to the persistence of HBV infection. Therefore, RNA inhibitors that target viral RNA for HBsAg reduction are considered an effective strategy to control HBV infection [99]. Preclinical studies in animals have shown that sequential therapy using a therapeutic vaccine, after HBsAg knockdown by siRNA, suppresses the viral load and increases the number of HBV-specific T cells and HBV neutralizing antibody production by releasing immune exhaustion in CHB [73].

Furthermore, a three-drug combination therapy consisting of a direct-acting antiviral drug and NA plus a host immune factor, RNAi (RG6346) + TLR7 agonist (RO7020531) + NA, RNAi (RG6346) + PEG-IFN + NA, CAM (RO7049389) + TLR7 agonist (RO 7020531) + NA have been investigated [74]. On the other hand, treatment with RNA inhibitors does not remove cccDNA and may cause a re-elevation of HBsAg after treatment [100]. Therefore, in actual clinical practice, combination therapy with multiple agents is necessary, and attention should be paid to the sustained response.

### 3.2. Potential of NA Discontinuation as a New HBV Treatment Strategy

**Figure 2:**
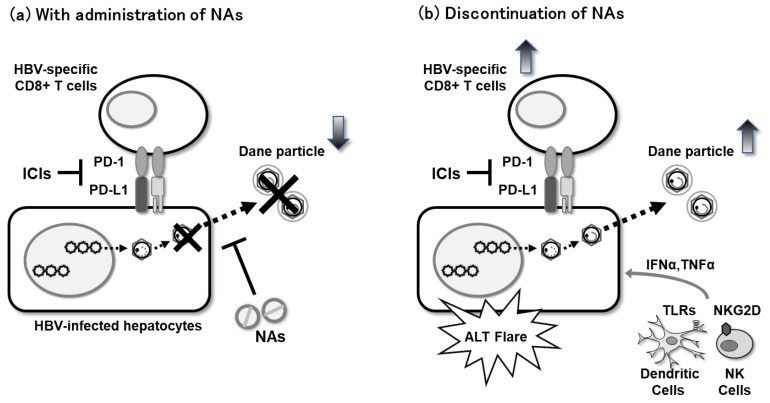
T cell responses during NAs administration. (**a**) With administration of NAs, HBV-specific CD8^+^ T cells remain unchanged, and ALT does not increase. (**b**) Without administration of NAs, HBV-specific CD8^+^ T cells are activated and ALT increases. Upward gradient arrows indicate an increase and downward gradient arrows indicate a decrease. ICIs, Immune checkpoint inhibitors.

Combination therapy with novel DAAs may lead to a functional cure. However, the rate of functional cure with siRNA, ASO alone is not sufficient, and immune induction is necessary. Discontinuation of NAs in CHB induces HBV re-replication, often considered an undesirable virological relapse, and ALT flares (Figure 2). HBV DNA reappears in 70% to 100% of patients who discontinue NAs, especially in HBeAg-negative patients, exceeding 2000 IU/mL at 12–24 weeks after ETV discontinuation [101,102].

On the other hand, this flare may be a desirable trigger for an immune response. In addition, immune reactivation therapeutic approaches, such as PD-1 inhibition or therapeutic vaccines, may have benefits when applied to patients undergoing NA discontinuation. Low HBsAg levels contribute to the prediction of response to NA discontinuation [103,104]. Therefore, a possible treatment concept is firstly to reduce HBsAg with the combination of NA with novel DAAs, such as siRNA, followed by NA discontinuation and sequential immune induction therapy. Because, in patients without cirrhosis, NA continuation seldom results in ALT flares during the treatment, NA discontinuation may cause hepatic failure and a fatal course after reactivation of CHB [105]. It is especially important to note that the possibility of death due to HBV reactivation after NA discontinuation has been observed in patients with pre-existing cirrhosis [106]. Distinguishing between treatment favorable and potentially unfavorable flares will be an important matter in the future.

### 3.3. The Role of HBcrAg in the Evaluation of Combination Therapy in Patients Receiving NA Therapy

Treatment with NAs is very good at inhibiting HBV-DNA replication, and therapy combining NAs with various other therapeutic agents currently in development will be necessary to achieve a functional cure, as described above. On the other hand, when determining whether a drug currently in development has an HBV DNA-lowering effect, HBV DNA is not an appropriate marker for assessing combination therapy because NA therapy masks the drug’s effect on HBV-DNA. However, it is not ethically appropriate to delay treatment with NAs for clinical trials. HBcrAg reflects the amount and transcriptional activity of cccDNA in hepatocytes and is not affected by NAs, making it suitable for monitoring the activity of CHB patients in NA combination therapy trials. This may contribute to the assessment of important drugs for future HBV therapy.

## 4. Conclusions

The goal of CHB therapy is the elimination of HBsAg and a functional cure. Many of the drugs currently in clinical trials and discussed in this review have shown good results in HBsAg reduction, which could not be easily achieved with PEG-IFN or NAs. In future drug development, it will be necessary to accumulate data showing a safety profile comparable to that of NAs. In particular, because few clinical trials have been conducted for cirrhosis B, it is expected that the indication for CHB patients without cirrhosis will be established first, followed by the accumulation of data for cirrhotic patients. In addition, the sensitivity to each agent may differ among HBV genotypes, which needs to be evaluated.

Finally, NAs and PEG-IFN remain the backbone of the multiple-drug combination therapies currently under development, and finding the best combination based on the characteristics of each drug will be important in therapeutic development.

## Figures and Tables

**Table 1:** Novel HBV therapeutic agents targeting HBV directly or indirectly, currently in phase II and beyond.

	Therapeutic Agents Targeting HBV Directly		
	Category or Target	Mechanism	Drug Name
(a)	cccDNA	Intended to destroy or repress HBV cccDNA	Preclinical studies only
(b)	Viral RNA	Silencing RNAs (siRNAs)	VIR-2218, RG6346, AB-729, JNJ-3989, ALG-125757, ARC-520, ARC-521, BB-103,
		Antisense Molecules (ASO)	Bepirovirsen(formerly IONIS-HBVRx), GSK 3228836, ALG-020572,
	RNA binding protein	RNA destabilizer	RG-7834
(c)	Core protein and capsid	Assembly inhibitor	Vebicorvir (ABI-H0731), EDP-514, JNJ-56136379, Morphothiadin,
(d)	HBsAg Inhibitors	Interferes with production of HBsAg	ALG-10133, REP-2139, REP 2165
(e)	Reverse transcription	Nucleos(t)ide Analogues	Lamivudine, Entecavir, Adefovir dipivoxi, Telbivudine, Tenofovir disoproxil, Tenofovir Alafenamide, Cledvudine (ATI-2173),
	FXR agonist	FXR agonist	ASC42, Vonafexor (EYP001)
Therapeutic Agents Targeting HBV Indirectly			
(f)	Viral entry	Interferes with HBV entry into liver cells	Myrcludex B (Bulevirtide, Hepcludex^®^), hzVSF (IgG4)
	FXR agonist	FXR agonist	ASC42, Vonafexor (EYP001)
	Interferons	Interferons	Interferon alfa 2b, Peginterferon alfa 2a
	Adaptive immune system	Therapeutic Vaccine	CVI-HBV-002, GS-4774, HerberNasvac, HepTcell, VBI-2601 (BRII-179), VTP-300, VVX001,
	Innate immune system	TLR-7 agonist	Vesatolimod (GS9620)
		TLR-8 agonist	Selgantolimod (GS9688)
	Exhausted T cell recognition	Checkpoint inhibitor	ASC22 (KN035 or Envafolimab)
	Neutralize HBV proteins	Monoclonal antibody	Lenvervimab (GC1102)

Hepatitis B Foundation: Drug Watch: https://www.hepb.org/treatment-and-management/drug-watch/ (accessed on 20 May 2022).
